# MRI evaluation of hypermobile lateral meniscus beyond distance measurements: the role of integrated popliteomeniscal fascicle assessment

**DOI:** 10.1186/s43019-026-00354-0

**Published:** 2026-10-23

**Authors:** Hyung Jun Park, Jung Hyun Park, Euddeum Shim, Se Rang Moon, Dong Hun Suh, Jae Gyoon Kim

**Affiliations:** 1 Department of Orthopedic Surgery, Korea University Ansan Hospital, Korea University College of Medicine, Ansan, Republic of Korea; 2 Department of Radiology, Korea University Ansan Hospital, Korea University College of Medicine, Ansan, Republic of Korea

**Keywords:** Hypermobile lateral meniscus, Popliteomeniscal fascicle integrity, Combined PMF score, MRI-based assessment

## Abstract

**Background:**

Hypermobile lateral meniscus (HLM) causes lateral knee pain and mechanical symptoms, but preoperative identification remains challenging because HLM is a dynamic abnormality that is not always captured on static magnetic resonance imaging (MRI). Previous MRI-based studies have mainly focused on linear measurements or qualitative popliteomeniscal fascicle (PMF) abnormalities. This study aimed to evaluate whether spatial MRI assessment beyond conventional linear distance measurements could help characterize arthroscopically confirmed HLM. We also aimed to determine whether MRI-derived markers could be organized into a clinically interpretable MRI-based approach to raise preoperative suspicion for HLM.

**Methods:**

This retrospective study included patients who underwent knee MRI followed by arthroscopic assessment of lateral meniscal stability. After propensity score matching, 53 knees with arthroscopically confirmed HLM and 53 non-HLM surgical controls were analyzed. MRI-derived variables included LM–popliteal tendon distance, region-of-interest-based measurement, MRI signal intensity ratio, and PMF integrity. Each PMF was evaluated separately and then summed to obtain the combined PMF score. Firth’s logistic regression, receiver operating characteristic curve, and Youden index analysis were performed.

**Results:**

Static measurements of LM–popliteal tendon separation did not differ between groups. In contrast, the sagittal MRI signal intensity ratio (odds ratio [OR], 1.067; 95% confidence interval [CI] 1.012–1.137; *p* = 0.044) and the combined PMF score were significantly associated with HLM (OR, 0.098; 95% CI 0.034–0.257; *p* < 0.001) were significantly associated with HLM in univariable analysis. In multivariable analysis, only the combined PMF score remained independently associated with HLM. The PMF score-only model showed an area under the curve of 0.794 (95% CI 0.709–0.879), with a Youden-derived threshold of ≤ 1.0, sensitivity of 58.5%, specificity of 86.8%, and accuracy of 72.6%.

**Conclusions:**

The combined PMF score was the MRI-derived marker most consistently associated with arthroscopically confirmed HLM. In this selected surgical cohort, lower combined PMF scores showed acceptable discrimination and relatively high specificity, although sensitivity was modest. Accordingly, a combined PMF score of ≤ 1.0 may provide adjunctive MRI information that raises suspicion for HLM when the clinical presentation is suggestive, but it cannot exclude HLM or independently establish the diagnosis, and the decision to perform arthroscopy should not be based on the score alone. Arthroscopic dynamic assessment remains necessary for definitive confirmation.

**Supplementary Information:**

The online version contains supplementary material available at https://doi.org/10.1186/s43019-026-00354-0.

## Introduction

Hypermobile lateral meniscus (HLM) refers to excessive mobility of the lateral meniscus (LM), which can cause lateral knee pain and mechanical symptoms such as locking [1, 2]. Rather than representing a single homogeneous disease entity, HLM may be better conceptualized as a spectrum of LM hypermobility confirmed by arthroscopic dynamic assessment [3–5]. Its shared pathomechanical feature is abnormal excursion of the LM, particularly the posterior horn [1–3, 6]. Recognizing HLM is clinically important because arthroscopic stabilization of symptomatic LM instability has been associated with improved patient-reported outcomes [2, 3, 7–10]. HLM may occur in isolation or in association with insufficiency of the posterolateral stabilizers [2, 3, 9–11]. The posterolateral stability of the LM is maintained by several structures, including the popliteomeniscal fascicles (PMFs), posterior capsule, and popliteus tendon complex [1–3, 6]. The PMFs are particularly relevant because they connect the LM to the popliteal tendon at the popliteal hiatus and restrain abnormal LM translation during knee motion [2–5]. Accordingly, PMF abnormality may serve as a structural MRI feature associated with LM hypermobility [2–5].

Despite its clinical importance, preoperative diagnosis of HLM remains challenging [2, 12, 13]. Magnetic resonance imaging (MRI) is widely utilized to evaluate intra-articular lesions; however, its ability to detect HLM remains suboptimal [3, 14, 15]. The anatomical complexity of the posterolateral corner can hinder clear visualization and contribute to variability across imaging protocols [6, 16]. Additionally, HLM is a dynamic disorder, whereas MRI captures the knee in a static position [13, 17, 18]. This mismatch may obscure mechanical abnormality, even when PMF attenuation or meniscocapsular insufficiency is present. Consequently, HLM may be missed unless the surgeon maintains a high index of suspicion based on clinical history, provocative maneuvers such as the figure-4 test, and targeted review of the posterolateral stabilizers [3, 14, 19].

Previous MRI-based studies have described findings associated with HLM, including widening or increased signal intensity of the popliteal hiatus [12, 14, 20]. However, it remains unclear which MRI-derived feature provides the most useful information for preoperative suspicion of HLM. Most objective assessments have focused on linear parameters such as the distance between the LM and the popliteal tendon [12, 14, 20]. These measurements may be limited because focal separation may not be consistently visualized on static MRI and may not capture the broader spatial configuration of dynamic instability [15, 21, 22]. Although spatial assessment beyond a single linear parameter may be conceptually useful, its clinical value has not been sufficiently investigated. Similarly, although individual PMF abnormalities have been associated with HLM, few studies have assessed the superior and inferior PMFs together as an integrated structural marker. A composite PMF-based assessment may therefore provide more clinically interpretable MRI markers.

This study aimed to evaluate whether spatial MRI assessment beyond conventional linear distance measurements could help characterize arthroscopically confirmed HLM. We also aimed to determine whether MRI-derived markers could be organized into a clinically interpretable MRI-based approach to raise preoperative suspicion for HLM. We hypothesized that spatial and PMF-related MRI assessment would be more consistently associated with HLM than static linear distance measurements. We further hypothesized that the most informative MRI-derived marker could provide adjunctive information for raising preoperative suspicion of HLM.

## Materials and methods

### Study design and population

This retrospective study analyzed patients who underwent arthroscopic knee surgery between December 2013 and December 2024. Surgical codes were first used to identify candidate patients for the HLM and non-HLM surgical control groups. Patients who underwent LM repair were screened as potential HLM cases, whereas patients without surgical codes for LM repair or meniscectomy were screened as potential non-HLM surgical controls. Because HLM is a dynamic arthroscopic diagnosis, group assignment was not based solely on surgical codes. Operative records, arthroscopic images, and surgical videos were reviewed in detail to confirm the presence or absence of abnormal LM hypermobility. HLM was defined as abnormal anterior translation of the LM posterior horn during arthroscopic probing, beyond the expected physiologic range [2, 20, 23]. This definition was intended to capture a clinically relevant spectrum of LM hypermobility or instability.

**Fig. 1:**
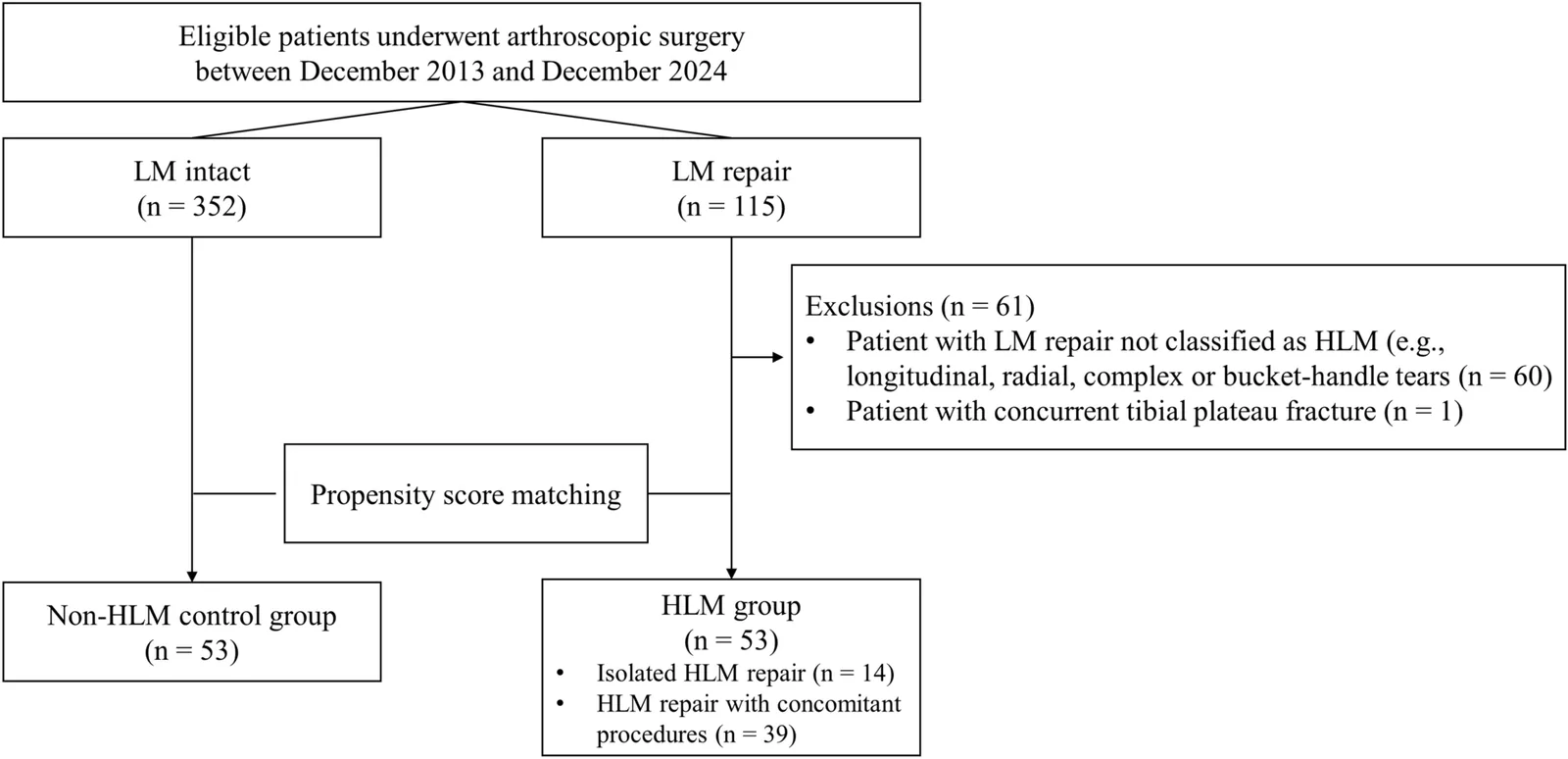
Patient selection for the study. The non-HLM surgical control group does not represent asymptomatic or healthy controls. Rather, it consisted of surgically treated patients who underwent arthroscopic assessment and were confirmed not to have HLM. *LM* lateral meniscus, *HLM* hypermobile lateral meniscus

Among the 115 knees initially screened from the LM repair group, repairs performed for intrameniscal tears rather than hypermobility were excluded. These included longitudinal, radial, complex, or bucket-handle tears of the LM (*n* = 60). One knee with a concomitant tibial plateau fracture was also excluded. After these exclusions, 53 knees were retained in the final HLM group. Among these, 14 knees underwent isolated LM stabilization, whereas 39 underwent HLM repair in conjunction with other knee procedures (Fig. 1). In patients undergoing isolated LM stabilization, arthroscopic evaluation was considered when the overall clinical presentation raised suspicion for symptomatic HLM, including persistent lateral-sided knee pain and/or mechanical symptoms, provocative examination findings such as the figure-4 test when documented, and supportive MRI findings such as widening of the popliteal hiatus when present. Arthroscopy was generally considered when symptoms persisted despite nonoperative treatment. In patients undergoing surgery for concomitant intra-articular pathology, LM stability was systematically assessed during arthroscopy, and additional LM stabilization was performed only when abnormal hypermobility was dynamically confirmed by probing. MRI findings alone were not used as an indication for arthroscopy or LM repair. The non-HLM surgical control group consisted of surgically treated patients in whom abnormal LM hypermobility was not observed during arthroscopic assessment. These patients were used as arthroscopically assessed non-HLM surgical controls rather than asymptomatic normal controls, because arthroscopic confirmation of a truly normal knee is not ethically or practically feasible. The initial control pool included 352 knees (Fig. 1).

**Fig. 2:**
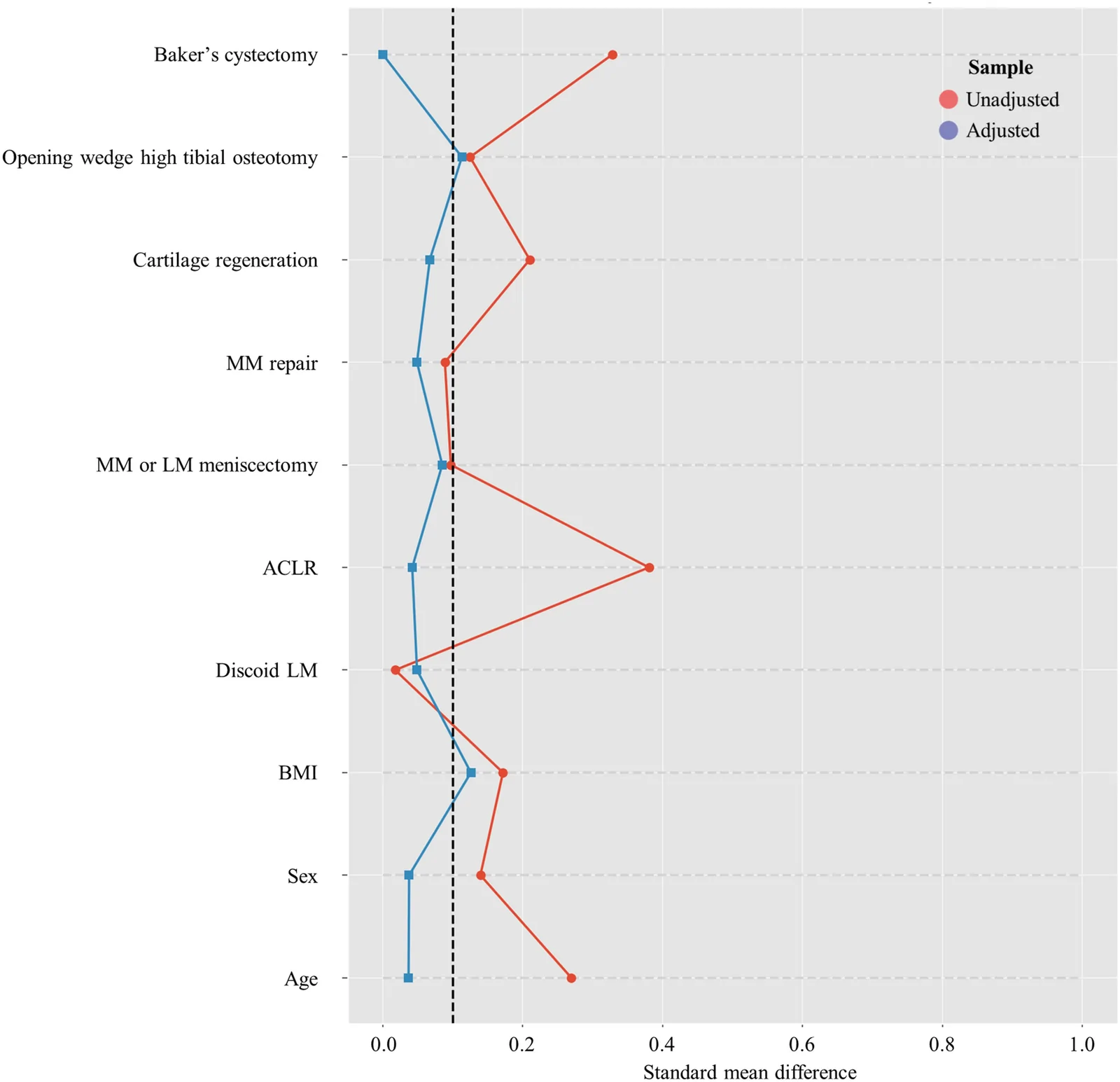
Standardized mean differences before and after propensity score matching. *MM* medial meniscus, *LM* lateral meniscus, *ACLR* anterior cruciate ligament reconstruction, *BMI* body mass index

Propensity score matching was performed to reduce baseline heterogeneity between the HLM cases and non-HLM surgical controls. Because this retrospective study was derived from patients undergoing various types of knee surgery rather than from a prospectively enrolled population with suspected HLM, substantial differences in demographic characteristics, underlying knee pathology, and surgical context were anticipated between groups. Matching was therefore used to create a clinically comparable surgical cohort for evaluating MRI-derived markers, rather than to estimate causal effects or population-level risk factors. Matching variables included age, sex, body mass index (BMI), presence of discoid LM, and types of concurrent surgeries. Age, sex, and BMI were included to reduce demographic heterogeneity, whereas discoid LM and concurrent procedures were included because they reflect differences in meniscal morphology, concomitant intra-articular pathology, and the underlying biomechanical or surgical context of the knee. By balancing these clinically relevant characteristics, we aimed to reduce major baseline differences between the surgical cohorts before evaluating MRI-derived markers associated with HLM. After 1:1 matching, 106 knees were included in the final matched analysis (Fig. 1). Covariate balance improved substantially after matching, with most SMDs below 0.1 (Table 1, Fig. 2).

**Table 1:** Baseline characteristics of patients according to the stability of the lateral meniscus before and after propensity score matching

	Non-HLM surgical control		HLM	SMD	
	Before matching (*n* = 352)	After matching (*n* = 53)	(*n* = 53)	Before matching	After matching
Age (years)	45.9 ± 16.6	40.6 ± 18.1	41.3 ± 17.9	0.269	0.037
Sex					
Female; *n* (%)	184 (52.3)	25 (47.2)	24 (45.3)	0.139	0.037
Height (cm)	162.9 ± 10.7	163.2 ± 14.0	165.7 ± 10.6		
Weight (kg)	68.8 ± 14.6	68.3 ± 15.9	68.4 ± 14.5		
BMI (kg/m^2^)	25.8 ± 4.2	25.3 ± 4.2	24.8 ± 4.0	0.172	0.126
Laterality					
Rt; *n* (%)	179 (50.9)	25 (47.2)	29 (54.7)		
Discoid LM; *n* (%)	64 (18.2)	9 (17.0)	10 (18.9)	0.018	0.049
Primary ACLR; *n* (%)	41 (11.6)	15 (28.3)	14 (26.4)	0.380	0.042
Meniscectomy; *n* (%)	72 (20.5)	15 (28.3)	13 (24.5)	0.097	0.085
Meniscus repair; *n* (%)	72 (20.5)	10 (18.9)	9 (17.0)	0.089	0.049
Opening wedge high tibial osteotomy; *n* (%)	23 (6.5)	1 (1.9)	2 (3.8)	0.125	0.113
Cartilage regeneration; *n* (%)	58 (16.5)	4 (7.5)	5 (9.4)	0.210	0.067
Baker’s cystectomy; *n* (%)	33 (9.4)	1 (1.9)	1 (1.9)	0.328	< 0.001

*n* number, *HLM* hypermobile lateral meniscus, *BMI* body mass index, *Rt* right, *LM* lateral meniscus, *ACLR* anterior cruciate ligament reconstruction

Values are presented as mean ± standard deviation or *n* (%)

SMD was calculated only for covariates included in the propensity score model (Methods)

### MRI evaluation

All patients underwent preoperative MRI examinations using a 3.0-Tesla machine (Achieva 3.0T and INGENIA ELITION X 3.0T, Philips, Netherlands; Skyra 3.0T, Siemens, Germany). To compare the HLM and non-HLM surgical control groups, we evaluated the spatial relationship between the LM posterior horn and the popliteal tendon by measuring linear distance and surface area within the region of interest (ROI). In addition, we quantified a fluid-sensitive marker using the T2-weighted signal intensity (SI) ratio and assessed the structural integrity of the PMFs. All measurements were performed using the hospital’s Picture Archiving and Communication System (PACS) software (INFINITT Healthcare, Seoul, South Korea).

**Fig. 3:**
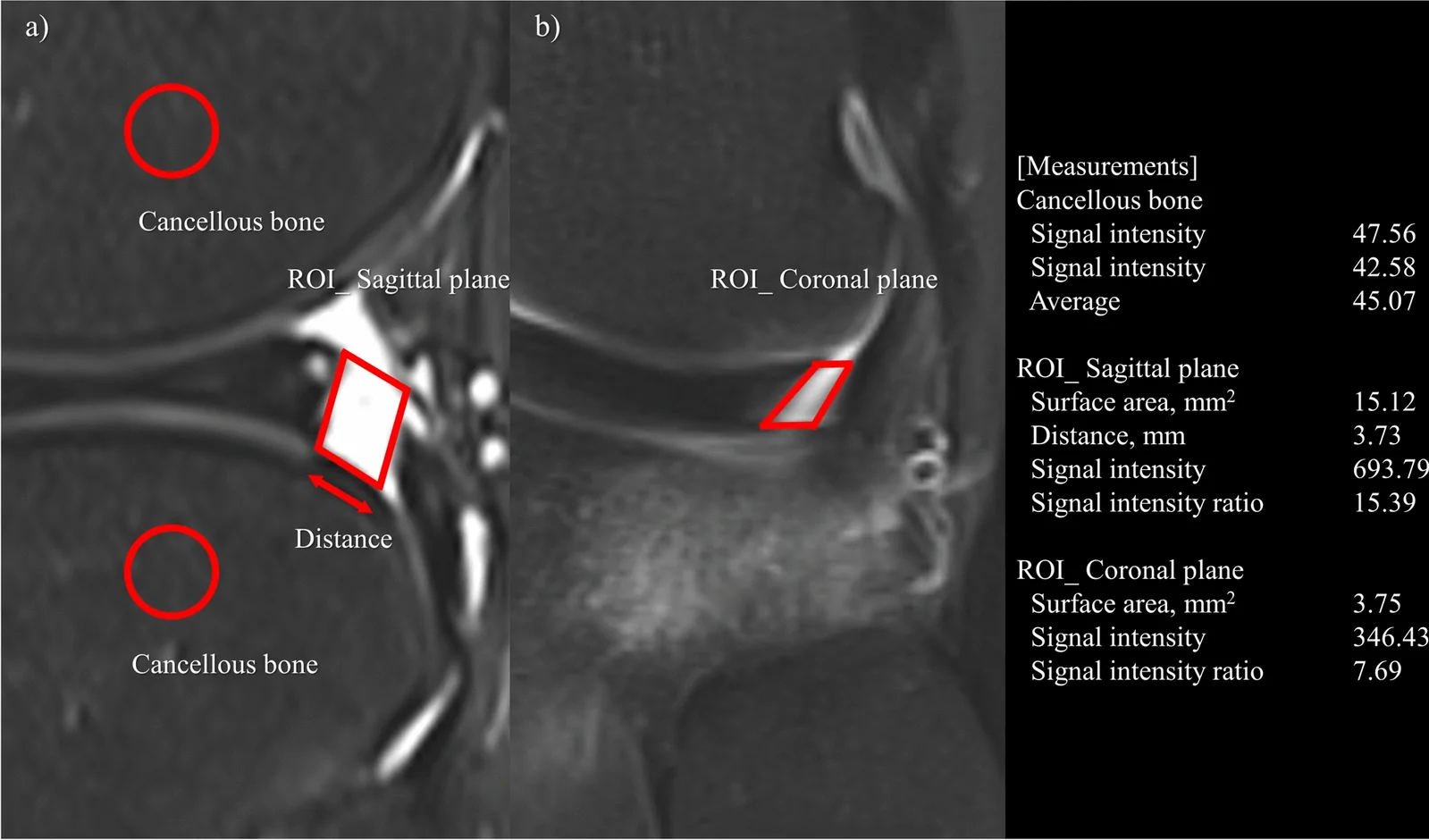
Measurement of the distance and region of interest between the lateral meniscus posterior horn and the popliteal tendon. **a** Sagittal plane image showing the distance (red arrow) and the ROI (red outline) defined between the LM posterior horn and the popliteal tendon. **b** Coronal plane image depicting the ROI (red outline) defined between the LM posterior horn and the popliteal tendon. ROI surface area and T2-weighted MRI signal intensity were measured, and the signal intensity ratio was calculated using cancellous bone as the internal reference. *ROI* region of interest, *LM* lateral meniscus

The distance between the LM posterior horn and the popliteal tendon was measured in the sagittal plane at the level where the popliteal tendon aligned with the lateral tibial plateau cartilage [17]. A reference line was drawn along the posterior margin of the LM posterior horn, with a parallel line through the popliteal tendon contact point. The shortest distance between these lines represented the degree of separation (Fig. 3a). However, recognizing that a single linear distance measurement may not fully reflect separation, an ROI was established to quantify the enclosed area [24–26]. ROI surface area was measured in sagittal and coronal planes. In the sagittal plane, the inferior boundary was drawn between the lowest points of the two parallel lines, aligned with the lateral tibial cartilage. The superior boundary extended from the highest enclosed region (Fig. 3a). In the coronal plane, the ROI was defined between the lateral margin of the LM and the popliteal tendon when both appeared in the same imaging plane (Fig. 3b). The superior and inferior boundaries of the ROI were determined by extending the corresponding LM margins to ensure a consistent and anatomically precise delineation of the enclosed region. To further characterize the degree of separation, T2-weighted MRI SI was measured within the ROI in both planes (Fig. 3) [25, 26]. Because raw MRI SI can vary according to scanner settings, acquisition parameters, and coil sensitivity, normalized SI ratios were calculated using internal reference regions [27–29]. The sagittal MRI SI ratio was calculated by dividing SI within the ROI by the mean SI of two arbitrary regions in the distal femoral condyle and proximal tibia cancellous bone [29]. This ratio was intended to reduce interpatient variability in raw SI and to provide a normalized fluid-sensitive marker of the LM–popliteal tendon interval.

**Fig. 4:**
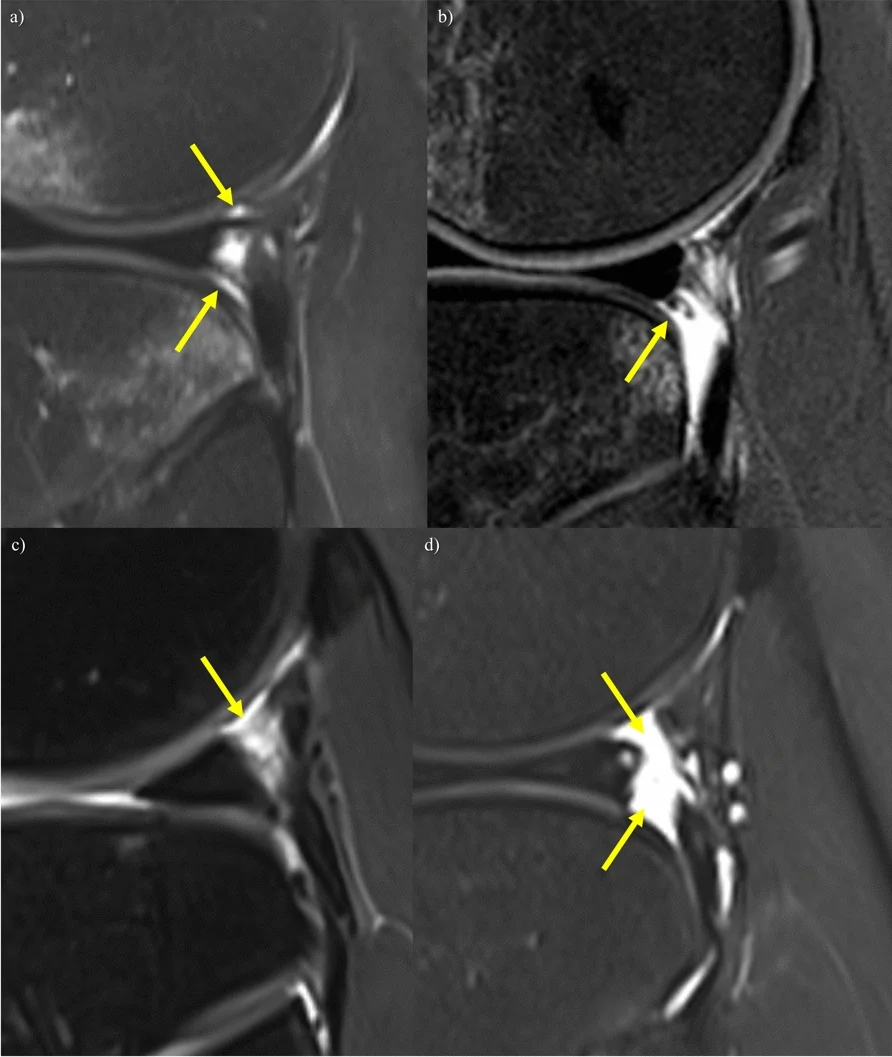
MRI-based classification of the popliteomeniscal fascicles. **a** Low-to-intermediate-signal bands representing both sPMF (upper yellow arrow) and iPMF (lower yellow arrow) are clearly visualized, classified as type I. **b** A low-to-intermediate-signal band of the iPMF (lower yellow arrow) is present but fails to reach the popliteal tendon, classified as type II. **c** The sPMF (upper yellow arrow) extends to the popliteal tendon but demonstrates a high-signal intensity, classified as type II. **d** Neither the sPMF (upper yellow arrow) nor iPMF (lower yellow arrow) is visualized, classified as type III. *PMF* popliteomeniscal fascicle, *sPMF* superior popliteomeniscal fascicle, *iPMF* inferior popliteomeniscal fascicle, *MRI* magnetic resonance imaging

PMF integrity was assessed on the sagittal MRI slices. Each PMF was visualized as superior and inferior bands adjacent to the posterior horn of the LM. These bands were classified based on their SI and continuity, using a modified version of the system proposed by Suganuma et al. [12]. The original description emphasized low-intensity bands; our study expanded the criteria to include low- to intermediate-signal bands to account for potential degenerative changes and variability in MRI appearance. To minimize confusion caused by inconsistent terminology in previous studies [1–3, 6], fascicles were designated as the superior and inferior PMF based on their relative position. Each fascicle was categorized into three types: type I, a low-to-intermediate-intensity band connecting the LM to the popliteal tendon (Fig. 4a); type II, a low-to-intermediate-intensity band that did not reach the tendon (Fig. 4b) or appeared poorly visualized with high SI (Fig. 4c); and type III, absence of a discernible band (Fig. 4d). For semiquantitative analysis, numerical values were assigned to each type (type I = 1, type II = 0.5, type III = 0). This ordinal scaling was chosen to reflect full, partial, or attenuated visualization and the absence of the fascicular band in a simple, clinically interpretable manner. The sum of the superior and inferior PMF values was defined as the combined PMF score. The combined PMF score was interpreted as an integrated structural MRI marker of PMF-related appearance rather than as a standalone definition of HLM.

All MRIs were independently reviewed by a musculoskeletal radiologist and a knee-specialized orthopedic surgeon who had not participated in any surgical procedures. Both evaluators were blinded to arthroscopic findings and clinical data during analysis.

### Surgical procedure

**Fig. 5:**
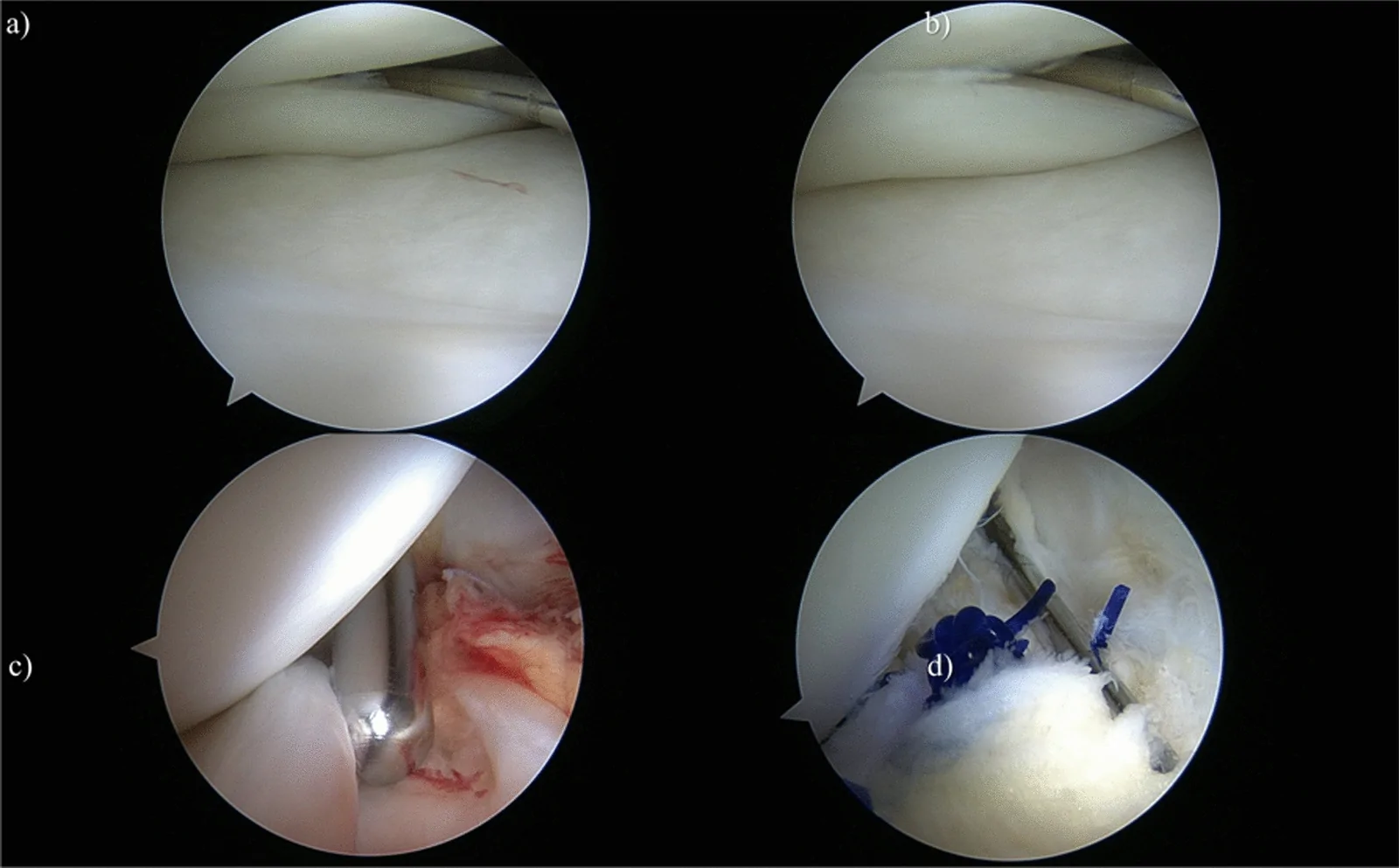
Arthroscopic confirmation and repair of hypermobile lateral meniscus. **a** Arthroscopic view of the LM posterior horn before probing. **b** Arthroscopic probing demonstrates abnormal mobility of the LM posterior horn. **c** Identification of the gap between the LM posterior horn and the posterolateral capsule. **d** Repair of the unstable LM posterior horn using an arthroscopic suture hook and absorbable sutures. *LM* lateral meniscus

A single senior surgeon performed all the arthroscopic procedures. The patients were positioned in a standard arthroscopic setup, and a systematic evaluation was conducted using either a 30° or 70° arthroscope. The anterolateral and anteromedial portals were utilized to facilitate the comprehensive assessment of intra-articular structures and to identify any associated lesions. LM posterior horn stability was assessed using an arthroscopic probe. If the posterior horn of the LM could be subluxated anteriorly beyond the apex of the lateral femoral condyle during arthroscopic probing, the patient was classified as the HLM group (Fig. 5a, b) [2, 20, 23]. Repair was performed using a location-directed strategy. The posterior horn was treated with a modified all-inside technique using a suture hook inserted through the posterolateral portal (Fig. 5c, d) [30]. Additionally, mid-body instability (posterolateral portion of LM, just anterior to the popliteal tendon) was repaired with a modified inside-out technique using a zone-specific cannula. All repairs were performed using no. 0 absorbable sutures (Ethicon, Somerville, New Jersey, USA) [23].

### Statistical analysis

Baseline characteristics were summarized before and after propensity score matching, and covariate balance was assessed using standardized mean differences (SMDs). For comparisons of MRI-derived variables in the final matched cohort, continuous variables with approximately normally distributed within-pair differences were analyzed using paired *t*-tests, whereas variables with skewed distributions were analyzed using Wilcoxon signed-rank tests. We evaluated the trend between the combined PMF score and the presence of HLM using a linear-by-linear association test. To visualize the relationship between the combined PMF score and the presence of HLM and examine its functional form, we applied a restricted cubic spline logistic regression model.

Firth’s penalized logistic regression analysis was used to identify MRI-derived markers associated with HLM [31]. Candidate MRI variables were first examined in univariable analyses. Variables with significant univariable associations were entered into a multivariable Firth logistic regression model. MRI variables that remained significant in the multivariable model were considered independent MRI markers associated with HLM. Model discrimination was assessed using receiver operating characteristic curve analysis, with the area under the curve (AUC) as the primary discrimination metric. To characterize the discriminative performance of the MRI marker retained in multivariable analysis, a single-marker Firth logistic model was fitted. This analysis was intended to quantify the marker’s association and discriminative characteristics rather than to establish a validated clinical prediction model. Its performance was compared with that of the multivariable MRI model using the DeLong test [32]. A cohort-specific threshold was determined using the Youden index, and sensitivity, specificity, and accuracy were calculated at this threshold. The Brier score was used to assess the overall prediction error, with lower values indicating better agreement between predicted probabilities and observed outcomes [33].

Internal validation was performed using 1000 bootstrap resamples [34]. For each bootstrap sample, the model was refitted, and its performance was evaluated both in the bootstrap sample and in the original matched cohort. Optimism was estimated as the mean difference between bootstrap-sample performance and test performance in the original cohort. Optimism-corrected AUC and Brier score were then calculated to assess discrimination and overall prediction error, respectively.

Inter-rater reliability for key MRI-derived measurements, including surface area, MRI SI ratio, and PMF classification, was assessed using intraclass correlation coefficients (ICC). All ICCs exceeded 0.85, indicating excellent reliability (SI ratio: 0.988 sagittal, 0.985 coronal; PMF classification: 0.974 for sPMF, 0.879 for iPMF). Baseline characteristics were analyzed using Statistical Package for the Social Sciences (SPSS) version 20 (SPSS Inc., Chicago, IL, USA). Regression analyses and DeLong tests were conducted using R version 4.4.1 (R Foundation for Statistical Computing, Vienna, Austria). A *p* < 0.05 was considered statistically significant.

## Results

### Factors associated with hypermobile lateral meniscus

MRI SI ratios and PMF-related MRI findings were associated with HLM, whereas static spatial measurements were not. No significant differences were observed in the distance between the LM and the popliteal tendon or in ROI surface area (Table 2). In contrast, the sagittal MRI SI ratio was higher in the HLM group than in the non-HLM surgical control group (median [interquartile range, IQR], 3.7 [2.3–7.8] versus 3.1 [2.5–4.9], *p* = 0.008). The coronal MRI SI ratio was also higher in the HLM group (2.7 [1.4–7.6] versus 2.6 [1.8–4.5], *p* = 0.013, Table 2).

**Table 2:** MRI findings in the hypermobile lateral meniscus and non-hypermobile lateral meniscus surgical control groups

	Non-HLM surgical control (*n* = 53)	HLM (*n* = 53)	*p*-value
LM–popliteal tendon distance (mm)	3.7 ± 1.6	3.6 ± 1.5	0.635*
ROI surface area (mm^2^)			
Sagittal plane	19.1 ± 8.6	19.3 ± 8.4	0.900*
Coronal plane	8.4 ± 7.1	8.8 ± 9.2	0.805*
Raw MRI signal intensity^†^			
Reference cancellous bone	54.5 [37.8–121.1]	53.7 [27.9–164.2]	0.418 ‡
Sagittal ROI	**234.2 [110.6–449.3]**	**361.8 [218.2–485.7]**	**0.017** ‡
MRI signal intensity ratio^†^			
Sagittal plane	**3.1 [2.5–4.9]**	**3.7 [2.3–7.8]**	**0.008** ‡
Coronal plane	**2.6 [1.8–4.5]**	**2.7 [1.4–7.6]**	**0.013** ‡
PMF integrity on MRI; *n* (%)			
Superior			< **0.001** §
Type I	**43 (81.1)**	**21 (39.6)**	
Type II	**10 (18.9)**	**17 (32.1)**	
Type III	**0 (0)**	**15 (28.3)**	
Inferior			**0.001** §
Type I	**34 (64.2)**	**14 (26.4)**	
Type II	**17 (32.1)**	**29 (54.7)**	
Type III	**2 (3.8)**	**10 (18.9)**	
Combined PMF score; *n* (%)			< **0.001** §
0	**0 (0)**	**4 (7.5)**	
0.5	**1 (1.9)**	**12 (22.6)**	
1	**6 (11.3)**	**15 (28.3)**	
1.5	**16 (30.2)**	**14 (26.4)**	
2	**30 (56.6)**	**8 (15.1)**	

Bold values indicate statistical significance (*P* < 0.05)

*MRI* magnetic resonance imaging, *HLM* hypermobile lateral meniscus, *n* number, *LM* lateral meniscus, *ROI* region of interest, *PMF* popliteomeniscal fascicle

Values are presented as mean ± standard deviation, median [interquartile range], or *n* (%), as appropriate

^†^ Skewed continuous variables are presented as median [interquartile range]

Statistical analysis: * Paired *t*-test; ‡ Wilcoxon signed-rank test; § linear-by-linear association test

**Fig. 6:**
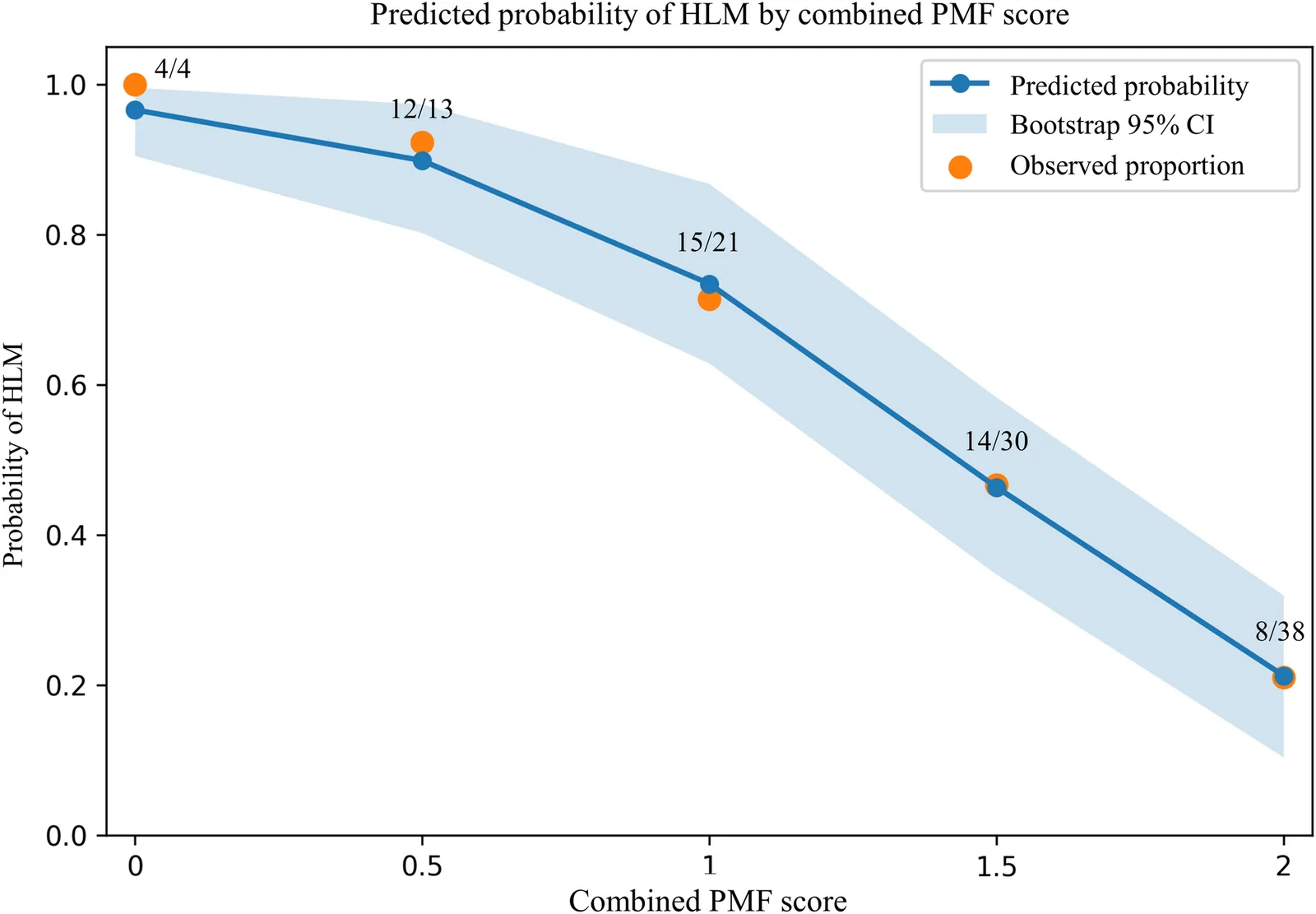
Observed and predicted probability of hypermobile lateral meniscus according to the combined PMF score. The solid line represents the predicted probability from the PMF score-only model, and the shaded area represents the bootstrap-based 95% confidence interval. Orange dots indicate the observed proportion of HLM within each combined PMF score category, with the corresponding number of HLM cases/total knees shown adjacent to each point. *CI* confidence interval, *HLM* hypermobile lateral meniscus, *PMF* popliteomeniscal fascicle

PMF integrity was significantly associated with HLM. Type III sPMF and iPMF tear findings were more frequent in the HLM group (28.3% and 18.9%, respectively) than in the non-HLM surgical control group (0% and 3.8%, respectively, Table 2). The combined PMF score also showed a significant inverse association with HLM (*p* < 0.001, Fig. 6). The observed proportion of HLM decreased progressively across combined PMF score categories: 100% (4/4), 92.3% (12/13), 71.4% (15/21), 46.7% (14/30), and 21.1% (8/38) for scores of 0, 0.5, 1.0, 1.5, and 2.0, respectively.

### Association and discriminative characteristics of the combined PMF score

The combined PMF score was the only independent MRI-derived marker associated with HLM in multivariable analysis. In univariable analysis, the sagittal MRI SI ratio was associated with HLM (odds ratio [OR], 1.067; 95% confidence interval [CI] 1.012–1.137; *p* = 0.044), and the combined PMF score showed an inverse association with HLM (OR, 0.098; 95% CI 0.034–0.257, *p* < 0.001, Table 3). When both variables were entered into the multivariable Firth logistic regression model, the combined PMF score remained significantly associated with HLM (OR, 0.106; 95% CI 0.040–0.281; *p* < 0.001), whereas the sagittal MRI SI ratio did not (Table 3). Thus, a single-marker Firth logistic model based on the combined PMF score was fitted to characterize its discriminative performance. The model was expressed as follows: Logit (*p*) = 3.344 − 2.327 × combined PMF score, where *p* represents the predicted probability of HLM. Predicted probabilities derived from this model are shown in Fig. 6.

**Table 3:** Univariable and multivariable logistic regression analysis of MRI-derived markers associated with hypermobile lateral meniscus

	Univariable analysis		Multivariable analysis	
	Odds ratio (95% CI)	*p*-value	Odds ratio (95% CI)	*p*-value
LM–popliteal tendon distance	0.945 (0.739–1.202)	0.642		
ROI surface area				
Sagittal plane	1.003 (0.959–1.049)	0.902		
Coronal plane	1.006 (0.960–1.054)	0.811		
MRI signal intensity ratio				
Sagittal plane	**1.067 (1.012–1.137)**	**0.044**	1.064 (0.987–1.148)	0.105
Coronal plane	1.058 (0.998–1.121)	0.058		
Combined PMF score	**0.098 (0.034–0.257)**	< **0.001**	**0.106 (0.040–0.281)**	< **0.001**

Bold values indicate statistical significance (*P* < 0.05)

Statistical analysis: Firth’s penalized logistic regression analysis. MRI variables significant in univariable analysis were entered into the multivariable model

Regression model with multivariable analysis: logit (*p*) = 2.853 + 0.062 × Sagittal MRI signal intensity ratio − 2.247 × combined PMF score

*LM* lateral meniscus, *MRI* magnetic resonance imaging, *ROI* region of interest, *PMF* popliteomeniscal fascicle

**Fig. 7:**
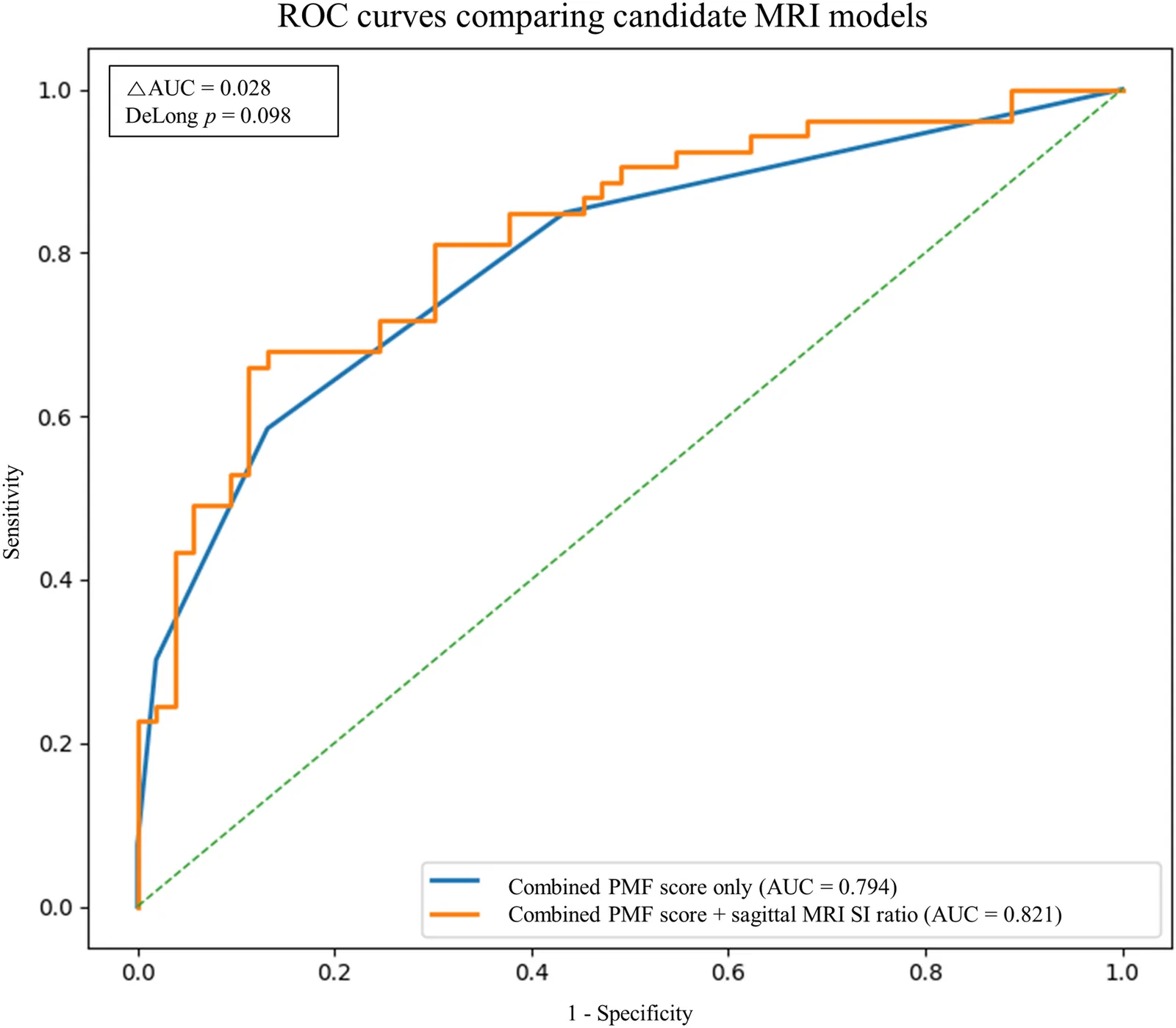
Receiver operating characteristic curves for the identification of hypermobile lateral meniscus. The PMF score-only model and the multivariable model showed AUCs of 0.794 and 0.821, respectively, with no significant difference on DeLong testing. The diagonal dashed line represents the reference line. *AUC* area under the curve, *MRI* magnetic resonance imaging, *ROC* receiver operating characteristic, *SI* signal intensity

The PMF score-only model showed discrimination comparable to the multivariable MRI model. The PMF score-only model showed an AUC of 0.794 (95% CI 0.709–0.879), sensitivity of 58.5%, specificity of 86.8%, accuracy of 72.6%, and Brier score of 0.179 (Table 4). The multivariable MRI model, which included sagittal MRI SI ratio and the combined PMF score, showed an AUC of 0.821 (95% CI 0.739–0.902), sensitivity of 66.0%, specificity of 88.7%, accuracy of 77.4%, and Brier score of 0.171 (Table 4). The increase in AUC was not statistically significant (ΔAUC, 0.028; 95% CI −0.005 to 0.061; *p* = 0.098) (Table 4, Fig. 7). Bootstrap internal validation indicated minimal optimism in the model’s discrimination. The optimism-corrected AUC was 0.794 for the PMF score-only model and 0.816 for the multivariable MRI model, while the optimism-corrected Brier scores were 0.185 and 0.180, respectively (Appendix 1). Using the Youden index, a combined PMF score of ≤ 1.0 was identified as the cohort-derived threshold. At this threshold, sensitivity was 58.5%, specificity was 86.8%, and overall accuracy was 72.6% (Table 4).

**Table 4:** Discriminative performance of the MRI-based models for identifying hypermobile lateral meniscus

	Variables included	AUC (95% CI)	Sensitivity (%)	Specificity (%)	Accuracy (%)	Brier score
Multivariable model	Sagittal MRI SI ratio + combined PMF score	0.821 (0.739–0.902)	66.0	88.7	77.4	0.171
PMF score-only model	Combined PMF score	0.794 (0.709–0.879)	58.5	86.8	72.6	0.179

Statistical analysis: DeLong test was performed to compare the two models (ΔAUC = 0.028; 95% CI −0.005 to 0.061; *p* = 0.098).

Regression model with PMF score-only: logit (*p*)  = 3.344 − 2.327 × combined PMF score.

Youden-derived threshold for the PMF score-only analysis: combined PMF score ≤ 1.0.

*AUC* area under the curve, *CI* confidence interval, *MRI* magnetic resonance imaging, *PMF* popliteomeniscal fascicle, *SI* signal intensity

## Discussion

The principal finding of this study was that the combined PMF score was the most consistent MRI-derived marker associated with arthroscopically confirmed HLM. Static measurements of LM–popliteal tendon separation, including linear distance and ROI surface area, did not adequately identify HLM. Sagittal MRI SI ratio was associated with HLM in the univariable analysis, but it was not independently significant after adjustment for the combined PMF score. Adding the sagittal MRI SI ratio to the combined PMF score produced only a modest and statistically nonsignificant improvement in AUC. These findings suggest that PMF-related structural assessment provides more clinically interpretable information than static, separation-based measurements in raising preoperative suspicion for HLM. However, the combined PMF score should be interpreted as an associated structural MRI marker rather than as a standalone definition of HLM.

Our findings partially supported the hypothesis that spatial assessment was associated with HLM, whereas static linear and area-based spatial measurements were not. Previous MRI studies have primarily focused on two domains: (1) the spatial relationship between LM and the popliteal tendon and (2) the structural integrity of the PMFs [12, 17]. Some studies have suggested that widening of the popliteal hiatus or increased LM–popliteal tendon separation may correlate with LM hypermobility or recurrent subluxation [14, 17]. However, these measurements are obtained from static MRI, whereas HLM is a dynamic pathology characterized by abnormal excursion of the LM posterior horn during knee motion [35, 36]. A single-frame linear measurement may therefore fail to capture episodic separations between the LM and the popliteal tendon [35]. In our study, LM–popliteal tendon distance was not significantly associated with HLM. Although the ROI-based assessment was introduced to evaluate spatial configuration beyond a single linear measurement, the ROI surface area did not differ between groups either. In contrast, the sagittal MRI SI ratio, a normalized signal-based parameter derived from the LM–popliteal tendon interval, was higher in the HLM group. This finding suggests that fluid-sensitive signal change within this interval may provide complementary information beyond static spatial size alone. Within this context, the integrated assessment of the PMFs appeared more informative. A previous study proposed a classification system that categorized each PMF by fascicular visibility, continuity, and signal characteristics; we adopted a modified version of this framework [12]. However, rather than appraising the superior and inferior PMFs in isolation, we synthesized both fascicles into the combined PMF score to provide a semiquantitative summary of PMF-related MRI appearance. This approach shifts the focus from single-frame spatial separation to fascicle-based structural assessment, while recognizing that PMF abnormality is not equivalent to HLM itself. The strong association between a lower combined PMF score and HLM supports the stabilizing role of the PMFs at the popliteal hiatus. Therefore, when HLM is clinically suspected, targeted MRI review of PMF morphology may provide useful adjunctive information, whereas arthroscopic dynamic assessment remains necessary for definitive confirmation.

Our findings partially supported our hypothesis that the most informative MRI-driven marker could provide adjunctive information for raising preoperative suspicion of HLM. Previous MRI-based studies have mainly focused on popliteal hiatus widening or qualitative PMF abnormalities, reporting only moderate diagnostic performance (AUC, 0.765; 95% CI: 0.644–0.887) [20]. These findings suggested that MRI may help raise preoperative suspicion, but they did not establish a definitive imaging-based diagnostic method for HLM. In our study, sagittal MRI SI ratio and combined PMF score were associated with HLM in univariable analysis. However, when both variables were entered into the multivariable regression model, only the combined PMF score remained independently associated with HLM. The combined PMF score-only model showed acceptable discrimination, with an AUC of 0.794, and the Youden-derived cohort-specific threshold was ≤ 1.0. At this threshold, the model showed modest sensitivity but relatively high specificity, suggesting that the combined PMF score may function better as a relatively specific, suspicion-raising MRI marker than as a screening tool to exclude HLM. However, because sensitivity was modest, a combined PMF score > 1.0 cannot be used to exclude HLM. Adding the sagittal MRI SI ratio increased the AUC from 0.794 to 0.821 and modestly improved sensitivity, accuracy, and the Brier score, but these improvements were not statistically significant. Therefore, sagittal MRI SI ratio may provide complementary signal-based information, but it should not be considered an essential component of the final MRI-based approach. Importantly, because this threshold was derived from a surgically treated cohort with arthroscopic confirmation, it should not be applied as a standalone screening criterion in general outpatient populations. Rather, a combined PMF score of ≤ 1.0 may help raise suspicion of HLM when clinical symptoms and physical examination findings are suggestive. However, the combined PMF score should be regarded as an exploratory, cohort-derived MRI marker, and the decision to perform arthroscopy should not be based on this score alone. Overall, these findings indicate that the combined PMF score may provide adjunctive MRI information when HLM is clinically suspected, while definitive diagnosis should still rely on arthroscopic dynamic evaluation of LM mobility.

This study had several limitations. First, it relied on static MRI, which may not have adequately captured the dynamic nature of HLM. HLM is characterized by abnormal LM mobility during knee motion or arthroscopic probing, whereas conventional MRI is acquired in a static position [13, 17, 18]. Advanced modalities, such as dynamic or weight-bearing MRI, may provide a more comprehensive evaluation of LM motion, but they were not available in this retrospective cohort. Second, HLM was evaluated as a spectrum of LM hypermobility or instability rather than as a single homogeneous disease entity. Although cases repaired primarily for ordinary intrameniscal tears were excluded to reduce tear-related pseudo-hypermobility, residual heterogeneity may remain. Therefore, the findings should be interpreted as applying to arthroscopically confirmed LM hypermobility or instability in a surgically treated cohort, rather than to a purely isolated congenital form of HLM. Third, the combined PMF score is an exploratory, cohort-derived semiquantitative MRI marker based on fascicular visibility, continuity, and signal characteristics. The 1, 0.5, and 0 weighting system provides a simple ordinal representation of PMF appearance but has not been externally validated. In addition, the modified PMF classification allowed low-to-intermediate-signal bands, which may limit direct comparability with prior classification systems. Although ICC was high in the present cohort, the reproducibility of this modified classification across institutions, MRI protocols, and readers remains to be established. The combined PMF score showed acceptable discrimination and relatively high specificity in this cohort, but its sensitivity was modest. Therefore, the score should be used to support preoperative suspicion rather than to screen for, exclude, or definitively diagnose HLM. Arthroscopic dynamic assessment remains necessary for confirmation. Fourth, this was a single-center study conducted over an extended period. Although MRI acquisition parameters were standardized where feasible, image review was performed independently under blinded conditions, and inter-rater reliability was excellent, temporal variation in imaging protocols or interpretation cannot be completely excluded. Although bootstrap internal validation indicated minimal optimism in model discrimination, external validation was unavailable. Prospective multicenter studies using independent cohorts are needed to confirm the reproducibility and generalizability of the PMF score-only model. In addition, MRI examinations were obtained using scanners from different vendors, which may have introduced residual measurement variability despite normalization of signal intensity ratios and blinded image review. Finally, this study was based on a selected surgical cohort and did not include a truly normal control group. The HLM group included both patients who underwent isolated stabilization for clinically suspected HLM and those in whom HLM was confirmed during arthroscopy performed for concomitant knee pathology. Because of the retrospective design, detailed preoperative documentation of symptom duration, provocative examination findings, and prior nonoperative treatment was not uniformly available. This heterogeneity in the clinical indication for arthroscopy may have introduced selection bias. Because arthroscopic confirmation cannot be ethically obtained in asymptomatic individuals, we used arthroscopically assessed non-HLM surgical controls. Propensity score matching was used to reduce major differences in demographic characteristics and concomitant knee pathology between the surgical cohorts; however, residual or unmeasured heterogeneity cannot be excluded. Moreover, the resulting matched cohort does not reflect the natural distribution of clinical characteristics among unselected patients evaluated for possible HLM, which may limit generalizability to the broader population.

## Conclusions

The combined PMF score was the most consistent MRI-derived marker associated with arthroscopically confirmed HLM. In this selected surgical cohort, lower combined PMF scores showed acceptable discrimination and relatively high specificity, although sensitivity was modest. Accordingly, a combined PMF score of ≤ 1.0 may provide adjunctive MRI information that raises suspicion for HLM when the clinical presentation is suggestive, but it cannot exclude HLM or independently establish the diagnosis, and the decision to perform arthroscopy should not be based on the score alone. Arthroscopic dynamic assessment remains necessary for definitive confirmation.

## Supplementary Information

Supplementary Material 1.

## Data Availability

The datasets generated and/or analyzed during the current study are not publicly available due to patient privacy and institutional restrictions, but are available from the corresponding author upon reasonable request and with appropriate approvals.
