# Supplementary material for: MRI evaluation of hypermobile lateral meniscus beyond distance measurements: the role of integrated popliteomeniscal fascicle assessment
Source: Knee Surg Relat Res. 2026 Oct 23;38:51. doi: 10.1186/s43019-026-00354-0 (PMC13695300; doi:10.1186/s43019-026-00354-0)
Supplement: Supplementary file 1 [file 43019_2026_354_MOESM1_ESM.docx]

Appendix 1. Bootstrap internal validation of MRI-based models

|  | Apparent AUC  (95% CI) | Mean optimism in AUC | Optimism-corrected AUC | Apparent Brier score | Mean optimism in Brier score | Optimism-corrected Brier score |
| --- | --- | --- | --- | --- | --- | --- |
| Multivariable model | 0.821 (0.739 – 0.902) | 0.006 | 0.816 | 0.171 | 0.009 | 0.180 |
| PMF score-only model | 0.794 (0.709 – 0.879) | -0.001 | 0.794 | 0.179 | 0.006 | 0.185 |

Statistical analysis: Internal validation was performed using 1,000 bootstrap resamples. Optimism was estimated as the mean difference between model performance in each bootstrap sample and performance of the bootstrap-derived model applied to the original matched cohort. Optimism-corrected AUC and Brier score were calculated to assess discrimination and overall prediction error, respectively, using internal validation.

Abbreviation: MRI, magnetic resonance imaging; AUC; area under the curve, CI; confidence interval; PMF, popliteomeniscal fascicle.
